# Effects of Environmental Agents of Denudation on Wetlands – A Tobit Regression Model Analysis

**DOI:** 10.21203/rs.3.rs-7442931/v1

**Published:** 2025-09-10

**Authors:** Abass Adeniyi Gazal, Carol J. Miller

**Affiliations:** Wayne State University College of Engineering; Wayne State University College of Engineering

**Keywords:** Nature-Based Solution, Run-off, Groundwater, Wetlands, Environment, Denudation, Tobit Model, Carbon Sequestering

## Abstract

Wetlands are vital ecosystems that play critical roles in biodiversity conservation, water purification, and climate regulation. However, they are increasingly vulnerable to environmental agents of denudation such as erosion, deforestation, and land-use change. Researchers have established the relationship between environmental agents, water-holding bodies, and hydrological cycle. It is imperative to state that this study intends to provide a quantitative comparison. Thus, this study employs a Tobit regression model to examine the effects of these agents on wetland size, using data from 51,885 observations and accounting for the censored nature of the wetland extent variable (Wetlevel). The model results indicate that several environmental factors significantly affect wetland size. Specifically, vegetation cover, soil moisture, and conservation efforts (erosion severity, conservation program presence, land cover heterogeneity) are positively associated with larger wetlands, while deforestation (deforestation rate) and erosion index exhibit strong negative association. Notably, erosion index had one of the most substantial negative coefficients (–0.00100, p < 0.01), confirming it is a major driver of wetland loss. The model also found that climatic variables (rainfall, temperature variability, elevation, distance to urban center) and socio-economic indicators (farming intensity, population density, land use pressure index) significantly influence wetland dynamics. The Tobit model’s fit statistics (AIC/N = 2.689; ANOVA-based fit = 0.0159) affirm its suitability for modeling censored ecological data. These findings highlight the urgent need for integrated environmental management strategies targeting erosion control, forest conservation, and sustainable land use to protect and restore wetlands.

## 1. Introduction

### 1.1 Rationale

The advent of climate change and the resulting unfavorable environmental conditions coupled with the effects of the ever-increasing population leading to urbanization and environmental pollution (air pollution, water pollution) all pose threats to the ecosystem. The inefficiencies of the deteriorating infrastructure to manage the constantly increasing levels of externalities that result from anthropogenic activities also poses threats to the environment and humans. These problems lead one’s thoughts back to nature for a solution. Thus, scientists have been exploring alternative approaches that more closely align with nature’s function.

The Nature Based Solution NBS concept was first explained by the European Commission in 2015, as “… actions inspired by nature to address ecosystem challenges” (Schanze, 2017). Wetlands are often called the “Earth’s kidneys” because of their capacity to filter pollutants and regulate water flow (Fang et al., 2022). Wetlands provide a range of unique functions linked to several ecosystem remediation pathways essential for biodiversity conservation, climate change mitigation, and human well-being (Syed Saquib et al., 2022) and supporting essential economic activities (crop production) (Hambäck et al., 2023). For example, they facilitate the regulation of hydrological cycles, including flood and drought control (Wu et al., 2022; Rojas et al., 2022); maintenance of soil moisture and groundwater generation (István Gábor Hatvani et al., 2022); purification of water (István Gábor Hatvani et al., 2022); regulation of air quality (Fang et al., 2022); nutrient cycling (Malerba et al., 2022); carbon sequestration (Kreplin et al., 2021). Having stated the benefits and functions of the wetland; there is global interest and efforts to preserve the entire size of naturally existing wetlands and to construct man-made ones in regions experiencing heat waves and drought. The size of the wetland and the volume of water it holds play an essential role in the general effectiveness of the wetlands. It is imperative to state that there are many interconnected factors that affect wetland ecosystem, functions, and its management. Some of these factors are precipitation, solar irradiation, topography, afforestation, vegetative cover, deforestation, human intervention, climate change, policy etc. Researchers around the globe have provided scientific literature which has assessed how these factors interact with wetlands. According to Dawson et al. (2003), wetland ecosystems depend on and are significantly impacted by water levels and changes in precipitation. Thus, a modelling assessment of water balance was undertaken by Dawson et al. (2003) for the UK and Ireland using current and future climate scenarios. Results showed that water availability could increase in winter across the whole region. Results from Dawson et al. (2003) also show that wetland habitat in East Anglia indicated that significant seasonal stresses could occur due to climate change and the associated lowering of water levels. Chen et al. (2019) assessed impact of wetland management and environmental protection plans (which are sometimes used interchangeably) on the wetland ecosystem. Chen et al. (2019) employed qualitative and quantitative measurements of wetland environments to create plan indexes using criteria. Thus, this study evaluates the real conditions and attributes DEMATEL (Decision-Making Trial and Evaluation Laboratory) technique to construct the Influential Network Relation Map (INRM). These techniques resulted in a model which can be used to explain interdependence. The results can also propose a gap improvement in the development of a sustainable development plan for the environment. Chatterjee et al. (2015) opined that assessing the conditions of a wetland entails considering the factors such as ecological, environmental, and socio-economic impacts which are unpredictable in nature. The study by Chatterjee et al. (2015) which was conducted in a National Park in India employed the Fuzzy Analytic Network Process [FANP] to assess the relative importance of different factor on environmental degradation, restoration, and conservation. Findings from Chatterjee et al. (2015) explain the relative levels of each factor’s influence on National Park’s ecological system. The results propose improving water supply, optimizing the land use structure, and strengthening wetland protection laws. Furthermore, a study was carried in Scania, Sweden by Salimi & Scholz (2021) aimed to investigate the effects of climate change on water quality in wetland ecosystems subject to water level management. Salimi & Scholz (2021) simulated the current and future climate scenarios (the last 30 years of the century) based on existing data and different regional climate models (RCM) of the Scania County. The results indicate that the impact of climate scenarios markedly differs for peatlands and constructed wetlands, exhibiting an interactive influence on the aggregated chemical variables. The warmest climate is associated with a higher water purification function for artificial wetlands; however, the warmest climate results to a lower water purification function and a subsequent deterioration of peatland (natural wetlands) water quality, even if subjected to water level management. A study by Ryu et al. (2022) study evaluates ecosystem diversity and species richness levels of a site over the past 3200 years. Multivariate analysis was used to identify the primary depositional processes for each stage and infer the proximate influencing factors. The findings indicate that early ecosystem shifts were controlled by the stages of the delta cycle, while more recent shifts are associated with anthropogenic activities and the effects of global climate change such as rising sea levels, and more intense tropical cyclones. Having carried out a comprehensive appraisal of existing literature, it is apparent that previous studies have carried out ecological evaluation in a fragmented manner and assuming that environmental factors are independent; however, these assumptions do not hold of real-world environmental problems. Past literature has also not given adequate quantitative analysis of the interrelationships between factors affecting ecological ecosystems. This creates the need to operationalize the interconnections between environmental agents of denudation and their effect on wetlands.

Thus, this research aims to provide an overview of the importance of wetlands to ecosystem sustainability. It also intends to integrate interrelated ecological factors using a statistical model to provide a quantitative analysis to help identify the statistically significant parameters impacting the wetland ecosystem. This is accomplished specifically by quantifying how environmental agents of denudation impact on the wetland size (wetland water level) using a Tobit regression model. At this juncture, it is imperative to give an overview of what a Tobit regression model is. The Tobit model, developed by James Tobin in 1958 (Tobin, 1958), is a type of censored regression model designed for conditions in which the dependent variable is either truncated or censored at a particular threshold (Greene, 2018). Unlike standard ordinary least squares (OLS) regression, which assumes a continuous and unbounded outcome, the Tobit model accounts for the possibility that observed outcomes are limited in range—such as when values fall below a certain observable limit (left-censoring) or above it (right-censoring). The Tobit model has been effectively applied in several fields including health economics, labor studies, transportation engineering, and environmental sciences (Agovino & Musella, 2020; Junk, 2012; Phuong et al., 2019; Chen et al., 2022; Amore & Murtinu, 2019; Lee, 2025).

The Tobit regression model is appropriate for this analysis because the dependent variable (wetland water level) has a natural lower bound at 0, i.e., it cannot be negative (−5 feet water is not possible). This makes it a left-censored variable. The Tobit model is also appropriate because it measures the factors influencing the magnitude of wetland size.

## 2. Methodology

### 2.1 Study Area and Data Collection

**Figure 1:**
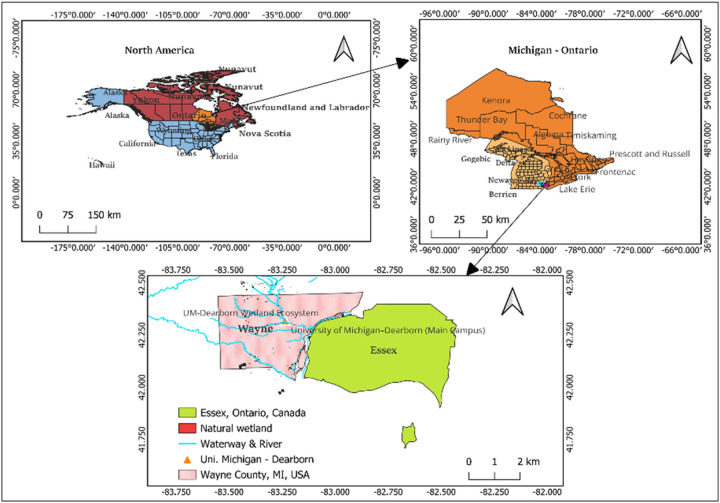
Map of the study site

To achieve the objectives of this research, primary data was collected and analyzed; this section gives a comprehensive methodological context. This study was conducted within the framework of a wetlands monitoring project based on the campus of the University of Michigan, Dearborn, with fieldwork covering the surrounding wetland ecosystems in Southeast Michigan (NFWF-NCRF, 2025). The region – shown on Fig. 1 - was selected due to its ecological significance and observed vulnerability to environmental degradation processes such as erosion, deforestation, and hydrological disruptions.

**Figure 2:**
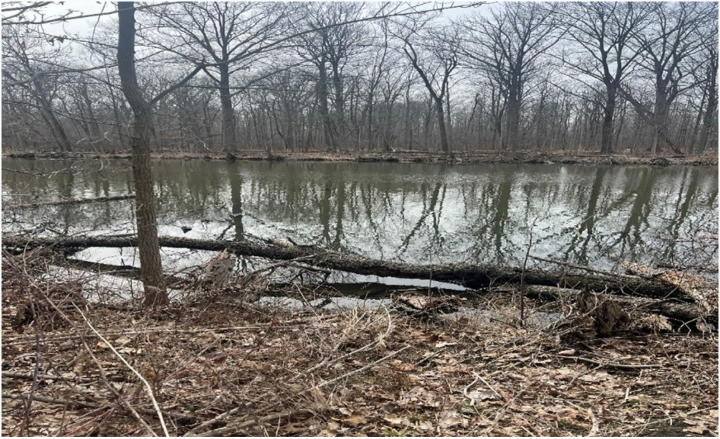
Wetland ecosystem study site at University of Michigan, Dearborn (March 27^th^, 2025)

**Figure 3:**
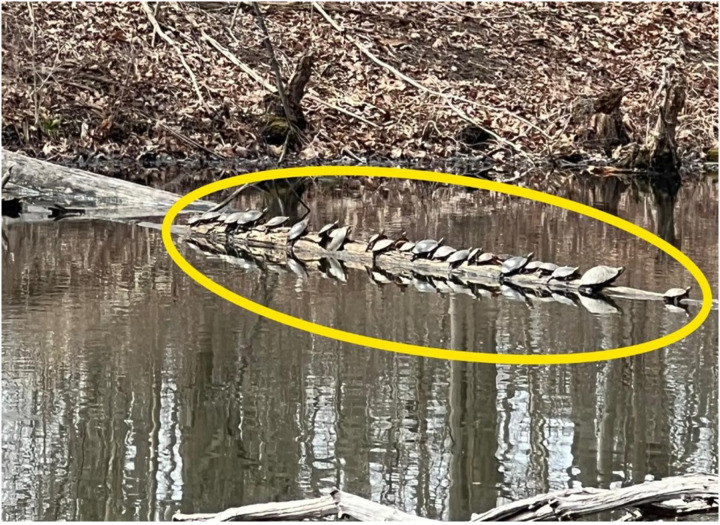
Study site (a thriving ecosystem where turtles, birds, shrubs, and fish are flourishing) (March 27^th^, 2025)

Primary data were collected over a 9-month period, from March 2024 to December 2024 (NFWF-NCRF, 2025). A combination of direct field measurements structured environmental assessments, and geo-referenced survey tools (e.g., GPS mapping, drone imaging, and ground-truthing) was used to obtain comprehensive data related to wetland extent and denudation variables. The sampling approach involved stratified random sampling to ensure coverage of the wetland and levels of anthropogenic influence. Figures 2 and 3 show the study site, while on a research team visit.

### 2.2 Variables and Measurement

The dependent variable in the analysis is Wetlevel, representing the spatial extent or size of wetland areas in square meters, as measured and geo-coded during the field survey. Due to instances where certain plots registered zero wetland presence (e.g., due to full degradation or seasonal drying), the variable is treated as left-censored at zero.

Seventeen independent variables (V1 to V17) were recorded, representing environmental agents and contextual factors associated with denudation. These include:

- V1–V3: Climatic and hydrological indices (e.g., average rainfall, soil moisture, water table depth)
- V4–V6: Denudation indicators (e.g., deforestation rate, slope gradient, erosion severity)
- V7–V11: Land-use and conservation management metrics (e.g., vegetation density, presence of conservation programs, land cover type)
- V12–V14: Geospatial and topographic characteristics (e.g., elevation, proximity to urban areas)
- V15–V17: Socio-economic and anthropogenic activity variables (e.g., farming intensity, population density, land-use pressure index). Table 1 below provides a list of all variables along with a description and data type for each.

### 2.3 Data Analysis and Model Specification

Given the censored nature of the dependent variable (with a significant proportion of observations at zero wetland presence), a Tobit regression model was employed. The Tobit model is appropriate when the outcome variable is continuous but constrained —here, wetland size cannot fall below zero, which violates the assumptions of ordinary least squares (OLS) regression. All variables were cleaned, normalized - using python and excel - where necessary to avoid data bias and ensure uniformity (e.g., log transformation for skewed distributions). Several iterations and combinations of independent variables were applied. These iterations helped to identify the significant variables from the list of 16 independent variables. The estimated coefficients of the model were rounded up to three significant figures to enhance clarity and interpretability. Variables with negligible coefficients – suggesting limited clarity for variations in the dependent variable – were subsequently excluded in later model iterations to improve overall goodness of fit.

## 3. Results

The Tobit model was employed to estimate the effects of environmental and anthropogenic variables on wetland size (Wetlevel), accounting for the censored nature of the dependent variable. The first iteration had 5 variables (V1, V2, V3, V4, V5) in its code (available in the Appendix). The iterations performed provide Log likelihood and Akaike Information Criterion (AIC). Log likelihood is a key metric used in Maximum Likelihood Estimation (MLE) commonly used in Tobit, probit, logit, and several other statistics (Greene, 2018; Verbeek, 2017). The likelihood of a model refers to the probability of observing a given data. The log likelihood is simply the natural logarithm of that likelihood function. The log likelihood value tells us how well the model fits the data (a higher (less negative) log likelihood means the model fits the data better while a lower (more negative) log likelihood means the model fits the data worse) (Wooldridge, 2010). The Akaike Information Criterion (AIC) is also another widely used statistic for model selection. It balances model fitness and complexity to help determine which statistical model is best suited for a given dataset (Wooldridge, 2010). The results of the first iteration are as follows: Log Likelihood value of −74,727.42; a higher (less negative) value means a better model fit. AIC value of 149,466.8 a lower AIC value indicates a better trade-off between fitness and complexity.

More iterations followed the first iteration to determine the significant parameters and model of best fit. The final iteration and analyses were based on 51,885 observations, yielding a log-likelihood value of − 69,735.48 and an Akaike Information Criterion (AIC) of 139,505.0 (AIC/N = 2.689), suggesting good model fit for limited dependent variable data and confirming the appropriateness of using a censored regression approach. Tables 2 and 3 summarize the estimated coefficients, standard errors, z-statistics, p-values, and 95% confidence intervals for each variable after the first and final iteration, respectively.

### 3.1 Interpretation of Model Parameters

This section discusses the estimated coefficients from the Tobit model, interpreting their direction, magnitude, and statistical significance in the context of wetland size dynamics. The statistical significance of each independent variable is assessed to determine whether it has a meaningful effect on the dependent variable (Greene, 2018). Every variable in the Tobit output has a corresponding p-value. Therefore, a p-value < 0.05, indicates that the variable is statistically significant at the 5% level. If p-value < 0.01, it is highly significant, and if the p-value > 0.10, it is not statistically significant (Greene, 2018). All interpretations are based on a 95% confidence level. Model estimation was conducted using Maximum Likelihood Estimation (MLE). The estimation yielded robust results with a log-likelihood value of − 69,735.48 and an AIC/N ratio of 2.689, indicating good model fit.

Vegetation density and soil moisture are the most powerful positive predictors of wetland extent. Erosion index and deforestation rate are the strongest negative predictors, clearly illustrating the destructive role of denudation. Interestingly, some socio-economic variables (farming intensity, population density, land use pressure index) showed positive effects, possibly due to managed land use or conservation efforts near population centers. A comprehensive explanation of how each variable impacted the overall model is discussed below.

#### 3.1.1 Climatic and Environmental Variables

The analysis revealed that climatic and environmental variables significantly influence wetland size, indicating the complex interconnectedness between hydrological inputs, vegetative cover, and denudation forces. Rainfall (V1) was found to have a positive and statistically significant at the 1% level (coefficient = 0.0000240), confirming its essential role in sustaining wetland ecosystems by contributing to the hydrological balance. This also aligns with hydrological theory, as greater rainfall enhances water input into wetland basins, thereby supporting wetland expansion or maintenance. The vegetation density measured using NDVI (V2) showed the strongest positive impact (coefficient = 0.08862, p < 0.01). This indicates that wetlands surrounded by or containing dense vegetation are more resilient to denudation processes. Vegetations (with the help of their roots) reduce runoff, stabilize soil, and facilitate groundwater recharge, all of which are beneficial to wetland integrity. This is similar with Paterson et al. (2023) who employed remote sensing data to show that vegetative biomass and canopy cover are positively associated with wetland surface area due to evapotranspiration control and soil stability. Similarly, soil moisture (V3) also had a significant positive coefficient (0.01455, p < 0.01) revealing that soil moisture plays a crucial role in maintaining wetlands. Areas with higher water retention in soils are more likely to support wetland conditions, either by reducing evaporation loss or enhancing subsurface water availability. Comparable results are reported by Tiner (2003) who emphasized the critical role of hydric soils in defining wetland hydrology.

Conversely, deforestation rate (V4) had a significant negative contribution on wetland size (−0.00094, p < 0.01), indicating that deforestation leads to reduction in wetland size as forest removal accelerates erosion and disturbs local hydrological balance of the ecosystem. This result is in tandem with literature of Millennium Ecosystem Assessment by Reid et al. (2005) and Junk et al. (2012). Both references showed that forest cover loss leads to degraded wetlands due to disrupted surface water inflow and sediment overload. Slope (V5) presented a small but significant favorable effect (0.00030, p < 0.01), implying that moderate slopes may assist in directing surface water into wetland basins (depressions), helping to sustain wetland conditions. At this juncture, it is imperative to state that excessive slope can increase erosion, so this effect is context dependent. Interestingly, erosion severity (V6) was positively associated with wetland size (0.00773, p < 0.01), which may seem counterintuitive. While erosion is destructive, this finding may indicate that erosion-prone zones, especially those near water body may also collect sediment and water, forming transient or new wetlands. This effect could be attributed to the accumulation of sediment and water in low-lying erosion-prone zones, potentially fostering temporary or emergent wetlands. Further spatial analysis may be needed to disentangle direct vs. indirect effects. In contrast, the erosion index (V10) exhibited a significant negative effect (−0.00100, p < 0.01), indicating that long-term or chronic erosion diminishes wetland area by degrading the soil’s structure and its capacity to retain water. This result aligns with results from Mitsch and Gosselink (2015) which gives account of how sedimentation from upstream erosion may temporarily expand wetlands. Together, these findings emphasize the delicate balance between environmental inputs and disturbances in shaping wetland dynamics, highlighting the critical need for integrative land and water management strategies in peri-urban ecosystems.

#### 3.1.2 Conservation and Land Use Indicators

The influence of conservation efforts and land use patterns on wetland size is clearly reflected in the model results. The presence of conservation programs (V7) has a positive and statistically significant coefficient (0.00235), suggesting that wetlands situated within or near areas under active conservation intervention are more likely to retain or even expand in size. These programs implement erosion control measures, reforestation, hydrological management, and consistent ecological monitoring, all of which contribute to improved wetland health and resilience. Literature by Zedler and Kercher (2005) have demonstrated that active conservation interventions significantly restore hydrology and vegetation in previously degraded wetland ecosystems. Similarly, temperature variability (V9) exhibits a significant positive effect (0.00100), indicating that wetlands in regions with fluctuating temperatures may be more robust or resilient. This could be due to the adaptive capacity of native vegetation species or microclimatic buffering that allows wetlands to persist despite heat stress, although the magnitude of this effect is small. Land cover heterogeneity (V11) emerges as a particularly influential factor, with a large and significant positive coefficient (0.00845). This underscores the importance of a diverse landscape matrix—comprising forests, grasslands, agricultural plots, and transitional habitats—in maintaining hydrological balance and enhancing ecological connectivity. Such diversity may mitigate runoff, reduce erosion, and provide ecological corridors that sustain wetland dynamics. Finally, the distance from urban centers (V14) also has a significant positive relationship (0.00099) with wetland size, highlighting that wetlands located farther from densely built environments tend to be larger. This finding agrees with results from (Davidson, 2014) who argued that peri-urban wetlands (wetland located between urban areas and rural landscapes) face the highest risk from land conversion, while remote wetlands remain ecologically intact. This aligns with established findings on the adverse effects of urban sprawl, where infrastructure development, land conversion, and pollution encroach upon and diminish natural wetland systems. Together, these variables point to the importance of landscape planning, conservation zoning, and sustainable land use in protecting peri-urban wetlands from degradation.

#### 3.1.3 Socioeconomic and Anthropogenic Factors

The influence of socioeconomic and anthropogenic variables on wetland size reveals a complex interplay between human activities and environmental resilience. The model analysis results show that the elevation coefficient (V12) (0.00032) is small; however, it is significant. This suggests that elevation has a marginally positive effect on wetland size. It is likely that moderate elevation reduces flooding risk while allowing water accumulation in valleys or terraces conducive to wetlands. The coefficient of the farming intensity variable (V15) is positive, which is unforeseen as farming is often linked with encroachment and general environmental degradation. However, in the context of this study, it may reflect the prevalence of agro-ecological systems such as rice paddies or irrigation-based farming practices that maintain semi-aquatic conditions, effectively supporting wetland-like environments. Furthermore, this may indicate integrated land management approaches where agricultural practices are designed to coexist with wetland conservation strategies. The population density (V16) contributes to a small but significant positive effect (0.00104). It suggests that wetlands in densely populated regions may not necessarily be in decline; rather, they could be actively preserved due to their socio-ecological value—providing essential services such as flood mitigation, recreational spaces, or even community identity. In these densely inhabited regions. Wetlands are preserved either due to policy, awareness creation, or ecosystem service needs (e.g., flood control, recreation). It may also reflect wetlands integrated into green infrastructure. The land use pressure index coefficient (V17) is positive and significant, indicating that areas under active land use pressure may still retain wetlands, due to targeted management, zoning regulations, or ecological monitoring. This may reflect policy-driven resilience rather than natural equilibrium. This finding is corroborated by Mishra et al. (2021) which found that wetlands in urbanizing regions may persist and expand due to flood control infrastructure, stormwater retention, and policy-driven preservation in urban environment planning.

### 3.2 Insignificant Variable

Distance to Nearest Water Body: This variable was statistically insignificant (p = 0.487), indicating that proximity to other water bodies does not significantly determine wetland size in this context. Wetlands may be more influenced by local geomorphology and land cover than by spatial proximity to rivers or lakes.

## 4. Discussion

The results from the Tobit model estimation provide critical insights into how various environmental and anthropogenic variables affect wetland size in the study area. Statistically significant relationships, both positive and negative, confirm that wetland dynamics are influenced by a complex interplay of climatic, geomorphological, land use, and human-related factors. One of the most influential factors identified was vegetation density, measured through NDVI, which showed a strong positive relationship with wetland size. This aligns with ecological theory, as vegetative cover reduces surface runoff and erosion while enhancing groundwater recharge and maintaining soil structure, all of which contribute to the sustainability of wetlands (Dahanayake et al., 2024; Alderson et al., 2025; Tang et al., 2021). Soil moisture and rainfall also had positive and significant impacts, underscoring the importance of hydrological inputs in supporting wetland health. This supports findings from previous studies that emphasize the hydric nature of wetlands and their dependence on consistent water availability (Ferreira et al., 2023; Gupta et al., 2024; Mitsch and Gosselink, 2015). These climatic variables were critical in explaining wetland extent, reinforcing the idea that hydrological balance is the core foundation of wetland existence.

Conversely, deforestation exhibited a negative effect, illustrating how land cover changes can accelerate denudation processes and undermine wetland sustainability. The loss of tree cover often leads to soil destabilization, increased surface runoff, and sedimentation in wetland areas, which may reduce their size or lead to their conversion into drier land types. Interestingly, the erosion severity index had a significant positive coefficient, while the long-term erosion index showed a significant negative effect. This duality may point to the fact that while episodic erosion can create conditions for wetland formation in depression or low-lying areas, chronic erosion has a cumulative detrimental effect, degrading land and reducing its capacity to retain water. This nuanced relationship underscores the importance of differentiating between types and intensities of erosion in environmental modeling. The influence of socioeconomic and policy-related variables was also notable. For instance, conservation program presence was positively associated with wetland size, reinforcing the effectiveness of targeted management strategies in preserving or rehabilitating wetland ecosystems. Similarly, land cover heterogeneity showed a strong positive effect, highlighting the role of diverse landscapes in buffering against land degradation and supporting ecological resilience. Unexpectedly, variables such as farming intensity and population density also showed positive relationships with wetland size. This suggests that, in the context of the study area, human activities may coexist with or even support wetland ecosystems, through practices like sustainable agriculture, controlled irrigation (e.g., rice paddies), or integrated landscape planning. Alternatively, these findings might reflect localized management practices or the proximity of wetlands to productive agricultural land, necessitating further field investigation. Another noteworthy observation is the role of distance from urban centers, which was positively correlated with wetland size. This supports the hypothesis that urban expansion poses a threat to wetlands through land conversion, pollution, and altered drainage patterns, making remote wetlands more likely to remain intact. While most environmental factors had intuitive effects, distance to nearest water body was found to be statistically insignificant. This suggests that wetland dynamics in the study area are governed more by local topographical and land use factors than by proximity to rivers or lakes.

## 4. Conclusion

This study investigated the effects of environmental agents of denudation and anthropogenic factors on wetland size using a Tobit model approach. The results revealed a multifaceted set of influences, with vegetation cover, rainfall, soil moisture, and conservation presence serving as key positive drivers of wetland sustainability. In contrast, deforestation and chronic erosion were identified as significant threats to wetland integrity.

The analysis also highlighted some context-specific outcomes, including the beneficial associations between wetland size and population density, farming intensity, and land use pressure. These results suggest that under appropriate management and policy frameworks, human activity does not necessarily lead to environmental degradation and may, in some cases, support ecosystem functionality. The Tobit model proved effective in handling the censored nature of wetland size data, capturing both observed and latent relationships. From a policy perspective, these findings underscore the importance of integrating land use planning, erosion control, reforestation, and conservation initiatives in strategies aimed at protecting wetland ecosystems. Future studies should consider expanding the model to include interaction terms and spatial effects to better understand the localized dynamics of denudation. Moreover, incorporating long-term satellite data and hydrological simulations could enhance predictive accuracy and support adaptive management under changing climatic conditions.

## Supplementary Material

This is a list of supplementary files associated with this preprint. Click to download.

- Appendix.docx

## Figures and Tables

**Table 1:** Variable description and corresponding data types

Variable Code	Variable Description	Data Type
V1	Average monthly rainfall (mm)	Continuous
V2	Vegetation density index (NDVI score)	Continuous
V3	Soil moisture content (%)	Continuous
V4	Deforestation rate in surrounding buffer (%)	Continuous
V5	Ground slope angle (degrees)	Continuous
V6	Erosion severity score (scale: 0–10)	Ordinal
V7	Presence of conservation programs (1 = Yes, 0 = No)	Binary
V8	Distance to nearest water body (km)	Continuous
V9	Annual temperature variation (°C)	Continuous
V10	Erosion index (measured via sediment yield)	Continuous
V11	Land cover heterogeneity (Shannon index)	Continuous
V12	Elevation above sea level (m)	Continuous
V14	Distance to urban center (km)	Continuous
V15	Farming intensity index (crop cycles/year)	Continuous
V16	Population density (people/sq km)	Continuous
V17	Land-use pressure index (0–100 scale)	Continuous

**Table 2:** Variables and results after the first iteration

Variable description	Coefficient	Standard Error	z-value	p-value	95% Confidence Interval
Rainfall	0.00004237***	0.00000334	12.69	0.000	[0.00003583, 0.00004891]
Vegetation Density	0.07789***	0.00067	116.63	0.000	[0.07658, 0.07920]
Soil Moisture	0.01718***	0.00020	84.42	0.000	[0.01678, 0.01758]
Deforestation Rate	−0.000133***	0.000319	−41.82	0.000	[−0.00140, −0.00127]
Slope	0.00141***	0.000329	42.91	0.000	[0.000135, 0.00147]
Erosion Severity	0.00773***	0.00051	15.07	0.000	[0.00672, 0.00873]

* = Significant at the 10% level (p < 0.10)

** = Significant at the 5% level (p < 0.05)

*** = Significant at the 1% level (p < 0.01)

**Table 3:** Variables and results after the final iteration

Variable description	Coefficient	Standard Error	z-value	p-value	95% Confidence Interval
Rainfall	0.0000240***	0.0000031	7.78	0.000	[0.00001798, 0.00003007]
Vegetation Density	0.08862***	0.00069	129.33	0.000	[0.08728, 0.08997]
Soil Moisture	0.01455***	0.00023	62.27	0.000	[0.01409, 0.01501]
Deforestation Rate	−0.00094***	0.00031	−30.46	0.000	[−0.00100, −0.00088]
Slope	0.00030**	0.00010	2.96	0.003	[0.00010, 0.00049]
Erosion Severity	0.00773***	0.00051	15.07	0.000	[0.00672, 0.00873]
Conservation Program	0.00235***	0.00013	18.48	0.000	[0.00210, 0.00260]
Temperature Variability	0.00100***	0.00032	31.39	0.000	[0.00094, 0.00106]
Erosion Index	−0.00100***	0.00012	−81.91	0.000	[−0.00103, −0.00098]
Land Cover Heterogeneity	0.00845***	0.00052	16.28	0.000	[0.00743, 0.00946]
Elevation	0.00032**	0.00013	2.39	0.016	[0.00006, 0.00057]
Distance to Urban Center	0.00099***	0.00014	7.10	0.000	[0.00072, 0.00126]
Farming Intensity	0.00784***	0.00099	7.91	0.000	[0.00590, 0.00978]
Population Density	0.00104***	0.00014	7.49	0.000	[0.00077, 0.00131]
Land Use Pressure Index	0.01807***	0.00279	6.47	0.000	[0.01260, 0.02355]

* = Significant at the 10% level (p < 0.10)

** = Significant at the 5% level (p < 0.05)

*** = Significant at the 1% level (p < 0.01)

## Data Availability

The datasets analyzed in this study are available from the corresponding author on reasonable request.
